# Ex vivo efficacy of BCMA‐bispecific antibody TNB‐383B in relapsed/refractory multiple myeloma

**DOI:** 10.1002/jha2.69

**Published:** 2020-08-01

**Authors:** David M. Foureau, Manisha Bhutani, Myra Robinson, Fei Guo, Duy Pham, Ben Buelow, Nury Steuerwald, Katherine Rigby, Elise Tjaden, Marina Leonidas, Barry A. Paul, Shebli Atrash, Ami Ndiaye, James T. Symanowski, Peter M. Voorhees, Saad Z. Usmani

**Affiliations:** ^1^ Immune Monitoring Core Laboratory Levine Cancer Institute/Atrium Health Charlotte North Carolina USA; ^2^ Department of Hematologic Oncology and Blood Disorders Levine Cancer Institute/Atrium Health Charlotte North Carolina USA; ^3^ Department of Cancer Biostatistics Levine Cancer Institute/Atrium Health Charlotte North Carolina USA; ^4^ TeneoBio Menlo Park California USA; ^5^ Molecular Biology Core Laboratory Levine Cancer Institute/Atrium Health Charlotte North Carolina USA; ^6^ Hematology Oncology Translational Research Laboratory Levine Cancer Institute/Atrium Health Charlotte North Carolina USA

**Keywords:** antibody therapy, immunophenotyping, myeloma

## Abstract

TNB‐383B is a fully human BCMA‐targeting T‐cell engaging bispecific monoclonal antibody (T‐BsAb). We assessed ex vivo efficacy of this drug to mediate killing of bone marrow mononuclear cells (BMMCs) freshly isolated from 10 patients with relapsed multiple myeloma (MM). BMMC were treated ex vivo with TNB‐383B at doses ranging from 0.001‐1 μg. Plasma cell (PC) lysis, viability, BCMA expression, CTL distribution, and degranulation were assessed by flow cytometry. Cytokine response to TNB‐383B was quantified by multiplex protein assay. Dose‐dependent PC lysis was triggered in all cases by TNB‐383B at doses as low as 0.001 μg (*P* = .0102). Primary MM cells varied in BCMA expression. High BCMA^+^ PC count correlated with increased PC lysis (*P* = .005) and significant CTL degranulation specific to TNB‐383B treatment (*P* = .0153 at 1 μg). High E:T ratio in bone marrow specimens led to lower viable and higher apoptotic PC compared with low E:T ratio (*P* < .001). Three cytokines were significantly modulated by TNB‐383B: IL‐2/TNFα increased by ∼4 ± 3.5‐fold average (*P* < .005 at 1 μg) and IP10 increased by ∼50 ± 15‐fold (*P* < .001 at 1 μg). We conclude that TNB‐383B triggers primary PC lysis and CTL degranulation in a dose‐dependent fashion *ex vivo* with no T cell expansion and mild increase of CRS‐associated cytokines.

## 1 INTRODUCTION

B Cell maturation antigen (BCMA) is a cell surface protein selectively expressed on multiple myeloma (MM) cells, making it an ideal target for new therapeutic agents [1]. T‐cell redirecting therapies such as CAR‐T cells and T‐cell engaging bispecific antibodies (T‐BsAb) that target BCMA have shown excellent efficacy in relapsed/refractory MM (RRMM) in early‐phase clinical studies [2, 3, 4]. However, nonspecific over‐stimulation of T cells resulting in cytokine release syndrome (CRS) and neurotoxicity narrows the therapeutic window of these bio‐therapeutics [3, 5].

TNB‐383B is a next‐generation fully human bispecific monoclonal IgG4 antibody that has been tested in vitro using cell lines and mouse xenograft models, and is in clinical development for MM [6]. It consists of two heavy and one light chain(s) paired using knob‐in‐hole technology. Heavy chain 1 and the kappa light chain form the paratope that recognizes and binds with low affinity to human CD3, whereas heavy chain 2 is composed of two identical VH domains in sequence and targets BCMA with high affinity and avidity (*K*d ∼0.7 nM) [7]. The unique design of TNB‐383B maximizes MM target cell killing while reducing off‐target toxicity and uncontrolled cytokine release, thus improving its tolerability and efficacy.

In this study, we tested the ex vivo efficacy of TNB‐383B by utilizing bone marrow (BM) plasma cells (PC) and BM cytotoxic T lymphocytes (CTL) from patients with RRMM. By culturing BM mononuclear cells (BMMC) isolated from patients, we were able to (1) test TNB‐383B's ability to promote PC lysis and CTL degranulation, (2) assess cytokine responses induced by TNB‐383B, (3) determine the mechanism of TNB‐383B‐induced cell death ex vivo, and (4) identify tumor variables (BCMA expression) and tumor microenvironment variables (E:T ratio) potentiating TNB‐383B activity.

## 2 MATERIAL AND METHODS

### 2.1 Bone marrow samples

Patients with RRMM for whom BM aspirates were collected under an IRB‐approved specimen collection protocol were enrolled. Fresh BM aspirates were collected in K2‐EDTA tubes and delivered within 2 h of specimen collection to the Immune monitoring core laboratory for processing.

### 2.2 Preparation of BMMC

All reagents and buffers were warmed to room temperature prior to use. BM aspirates were treated with red blood cell lysis buffer (Santa Cruz, Dallas TX) for 15 min on a tube roller. Cell pellets were washed twice (350 × *g*, 5 min) in PC washing buffer (PBS pH 7.4, Ca/Mg free, 2% w/v BSA fraction V, 2 mM EDTA). Following RBC lysis, total nucleated cell count, and viability were assessed using Trypan blue exclusion assay (Sigma–Aldrich, St Louis, MO) to determine whether the specimen was suitable for in vitro assay (i.e. ≥25 million total nucleated cells, ≥95% viability). Cell pellets were re‐suspended in culture medium (IMDM, 10% v/v ultra‐low Ig FBS, 1% v/v, Glutamax, 1% v/v Penistrep) for Ficoll‐Paque density gradient separation (400 × *g*, 30 min, no break). After centrifugation, BMMC were washed once in PC culture medium (350 × *g*, 5 min). Following isolation, BMMC count and viability were assessed by Trypan blue exclusion assay to determine whether specimen was suitable for in vitro assay (ie, ≥10 million BMMC, ≥95% viability). Baseline PC content/viability, PC BCMA expression and effector to target ratio (E:T ratio; i.e. % CTLs divided by the %PCs) were assessed by flow cytometry (Table S1).

**Key points summary**

- TNB‐383B is a fully humanized T cell engager bispecific antibody targeting BCMA with a mild cytokine release profile
- TNB‐383B showed ex vivo anti‐myeloma activity against bone marrow aspirates from patients with relapsed multiple myeloma

### 2.3 T‐cell engaging bispecific antibodies

TNB‐383B (lot# OMTT086_Round5) and the negative control solely recognizing CD3 (lot# OMTT050_Round10) were formulated at a concentration of 2 mg/mL in PBS pH 7.4 and stored at −80°C. Material generated at TeneoBio was used in this study.

### 2.4 Ex vivo T‐BsAb assay

BMMCs were plated at a 1.25 × 10^6^ cell/mL density, in 200 μL PC culture medium, and treated with TNB‐383B or the negative control at four different doses: 0.001, 0.01, 0.1, and 1 μg. Each dose and experimental condition were run in duplicate. After 24 ± 2 h incubation at 37°C, 5% CO_2_, supernatants were harvested from the top of each sample well to measure cytokine‐release by BMMCs using Bio‐Rad human 27‐plex assay (Eotaxin, FGF basic, G‐CSF, GM‐CSF, IL‐1β, IL‐1ra, IL‐2, IL‐4, IL‐5, IL‐6, IL‐7, IL‐8, IL‐9, IL‐10, IL‐12, IL‐13, IL‐15, IL‐17, IP‐10, IFNγ, MCP‐1, MIP‐1α, MIP‐1β, RANTES, PDGF‐BB, TNF‐α, VEGF; Bio‐Rad, Hercules CA)

The cellular fraction was stained with a nine‐color antibody panel [CD45, CD38, CD138, CD45, CD3, CD8, CD269 (BCMA), CD107a, 7‐AAD, and Annexin V; see Table S1] by flow cytometry to determine E:T ratio (ie, CD8^+^ CTL:PC ratio), PC lysis, viability, BCMA expression, and CTL degranulation (Figure S1).

Percent PC lysis (%PC lysis) and CTL distribution (%CTL) were determined by normalizing average counts from each duplicate with the vehicle (untreated) control after 24 h (±2 h) incubation. Plasma Cell viability (% live PC) was determined by averaging the number of 7‐AAD negative cells from each duplicate. PC death by apoptosis (% apoptotic PC) was determined by averaging the number of 7‐AAD/Annexin V double positive cells from each duplicate.

BCMA expression was expressed as average frequency of PC expressing BCMA (PC BCMA^+^) and mean fluorescence intensity (BCMA MFI). CTL degranulation (% CD107a^+^ CTL) was determined by averaging MFI of CD107a expression by CTLs.

### 2.5 Flow cytometer set up and data output standardization

Flow cytometry data were acquired on a BD lsrFortessa (BD Biosciences San Jose CA). Compensation matrices calculation and data acquisition were performed using Diva v8 software (BD Biosciences). Flow cytometry data were analyzed using FlowJo version 10 software (FlowJo LLC).

To standardize data output, photomultiplier (PMT) voltages were adjusted monthly using eight‐peaks fluorescent beads (Spherotech, Lake Forest IL). Using the seventh peak, PMT voltages were adjusted to meet predetermined median fluorescence intensity (MFIs) on each fluorescence channel (Figure S2). The same beads were then tested monthly (Lot EAG01) to confirm MFIs remained within 5% of target values.

### 2.6 Statistical methods

Tukey's sequential trend test was performed for each measured variable, utilizing ANOVA models and contrast statements, to detect linear dose response trends to TNB‐383B or negative control treatments. Additionally, a parametric model (E_Max_) was used for each measured variable to estimate dose response curves, interpolating between tested doses. Two‐way factorial ANOVA was utilized to compare the main effects of E:T ratio (or PC phenotype) and dose level and the interaction effect between E:T ratio and dose level on measured variables.

## 3 RESULTS

BM aspirates were collected from 13 patients with RRMM, of which three failed QC due to low cell count, therefore 10 patients were included in final analysis. Disease characteristics and treatment details are shown in (Table 1). All patients had received at least three prior lines of therapy. Six were being actively treated at the time of BM specimen collection, four were off treatment (median 12.5 months, range 7‐40 months).

**TABLE 1:** Bone marrow specimen description

Specimen ID	Age/Sex	Disease^*^	Subtype	Prior Lines of Therapy	Latest line of treatment	Cytogenetic Risk^†^
PCD1020	66/F	MM	IgG‐K	3	Velcade maintenance	High Risk
PCD1201	66/F	MM	IgG‐L	3	RV maintenance(off therapy for 40mo)	High Risk
PCD1218	52/M	MM	IgG‐L	5	Elo‐Rd	High Risk
PCD1300	57/M	MM/PCL	IgA‐K	3	KRd followed by Mel200‐ASCT(off therapy for 13mo)	High Risk
PCD1421	59/M	MM	KLC	3	Velcade Maintenance(off therapy for 7mo)	High Risk
PCD1427	65/F	MM	IgG‐L	3	KRd‐Elo	Standard
PCD1441	71/M	MM	LLC	4	KRd‐Elo	Standard
PCD1447	68/F	MM	IgG‐K	4	CarCy(off therapy for 12mo)	High Risk
PCD1527^‡^	54/F	MM	KLC	3	Dara‐Vd	High Risk
PCD1542	53/M	MM	IgG‐K	6	Pom‐Dex	Standard

Abbreviations: MM: multiple myeloma Myeloma, PCL: plasma cell leukemia. Bor/V: Bortezomib, Car/K: Carlfilzomib, Dara: daratumumab, Dex/d: dexamethasone, Elo Elotuzimab, Mel200‐AST: high‐dose Melphalan and autologous stem cell transplant, Pom: pomalidamide R: Revlimid,

* All patients were relapsing at the time of bone marrow aspirate collection.

† [As per IWMG criteria] Standard Risk: normal cytogenetics, hyperdiploidy, or t(11;14); Intermediate Risk: [t(6;14), del 13, or others not in good or high risk; High Risk: del 17p, t(4;14), t(14;16), t(14;20), amplification 1q21, complex cytogenetics, or hypodiploidy.

‡ Plasma cells from this BMMC specimen were CD38 negative.

### 3.1 Bone marrow aspirates cell count and viability

BM aspirate pull numbers ranged from 1 to 9; five specimens were early pull (1‐3). Following RBC lysis, average cell counts were 65 ± 31 million nucleated cells at 98.4±1.2% viability. E:T ratio post‐density gradient separation ranged from 3:1 to 82:1 (median 4.8). BMA pull number was not a direct predictor of E:T ratio, indicating hemodilution did not impact the concentration of plasma cell/T cells. All specimens contained BCMA‐expressing PC (median 25.7%, range 7.7‐97.6%). In our cases, we did not see any association between BMMC E:T ratio or BCMA expression with number or type of prior therapies.

### 3.2 TNB‐383B induces PC lysis

We assessed the tumor lysis activity of TNB‐383B against patient derived MM cells. The BMMC were incubated with TNB‐383B or a negative control for 24 ± 2 h at doses ranging from 0.001 to 1 μg, followed by flow cytometry analysis to determine percentage of PC lysis, PC viability, and PC apoptosis.

**FIGURE 1:**
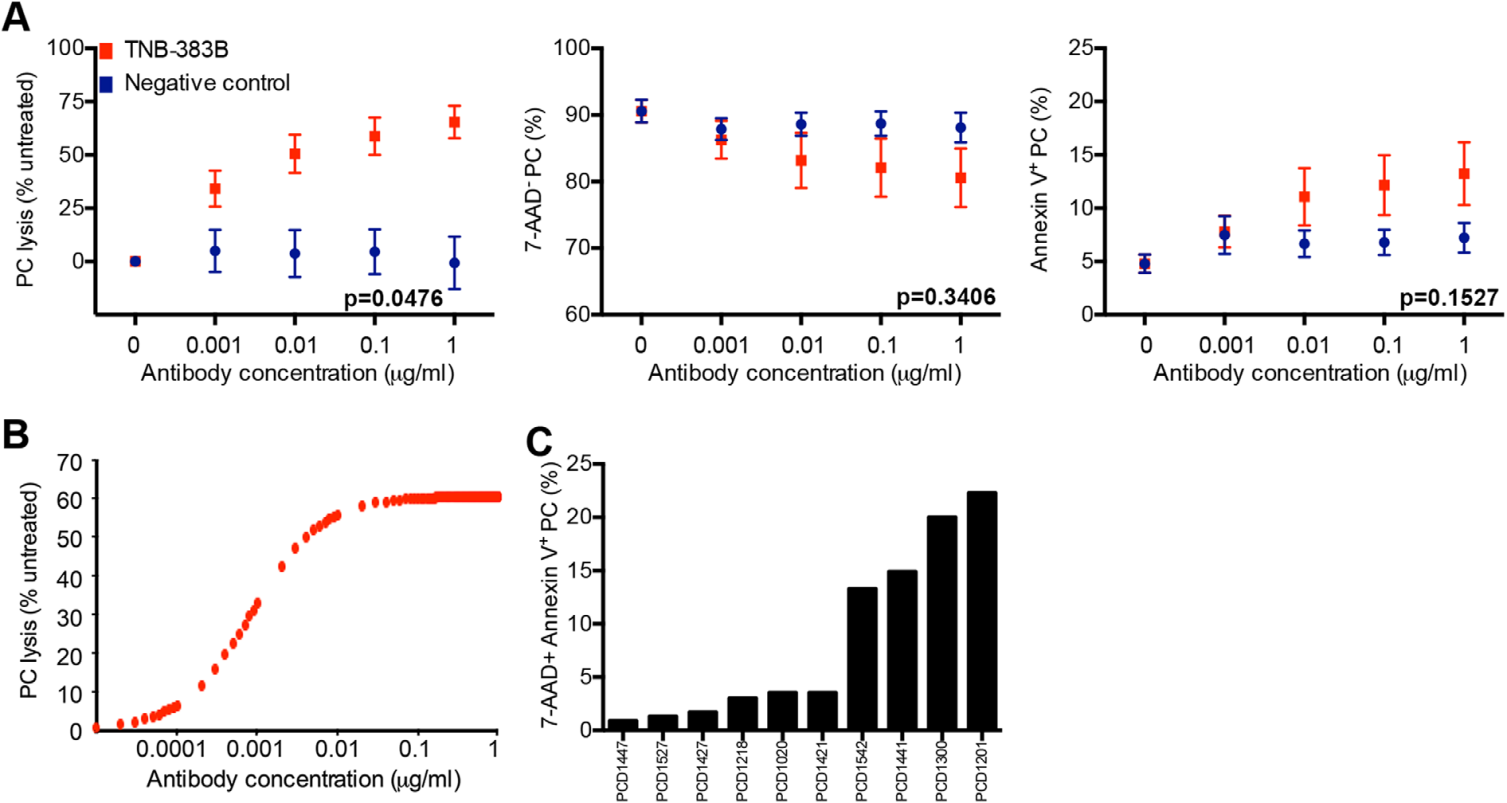
TNB‐383B anti‐plasma cell activity ex vivo. A, Interaction of treatment analysis to determine the impact of treatment (TNB‐383B vs. negative control) and dose (0.001 μg to 1 μg) on PC lysis, viability and apoptosis. Tukey's sequential trend test was performed for each experimental treatment group, and each variable tested, utilizing ANOVA models and contrast statements to detect a linear trend in experiments involving increasing doses. B, Dose‐dependent PC lysis induced by TNB‐383B using E_max_ parametric model to estimate dose response curves, interpolating between tested doses. C, PC apoptotic rate (% 7‐AAD^+^ Annexin‐V^+^) distribution among the 10 BMMC preparations tested following 24 ± 2 h ex vivo treatment with 1 μg/mL TNB‐383B

TNB‐383B induced autologous lysis of PC in all samples in a dose‐dependent manner, while the negative control was inactive (*P* = .0476; Figure 1A). TNB‐3835 activity was seen in all samples from heavily pretreated patients, including a patient (PCD1527) previously exposed to daratumumab. In our small sample size, we did not observe any association between TNB‐3835 activity and type of treatments and lines of therapies received by patients, or their disease stages. E_max_ parametric modeling showed that PC lysis was triggered by TNB‐383B at dose as low as 0.001 μg (Figure 1B).

Viability and apoptotic rate of remaining PC were not significantly different between TNB‐383B or negative control (Figure 1A). While the negative control did not induce significant changes in PC viability and PC apoptosis, the effect of TNB‐383B was highly heterogeneous. At the 1 μg T‐BsAb dose, PC apoptotic rate was above 10% (17.6±4.3%) for four specimens tested but it remained below 5% for the six remaining specimens (2.3 ± 1.2%; Figure 1C).

### 3.3 BCMA expression correlates with PC lysis by TNB‐383B

**FIGURE 2:**
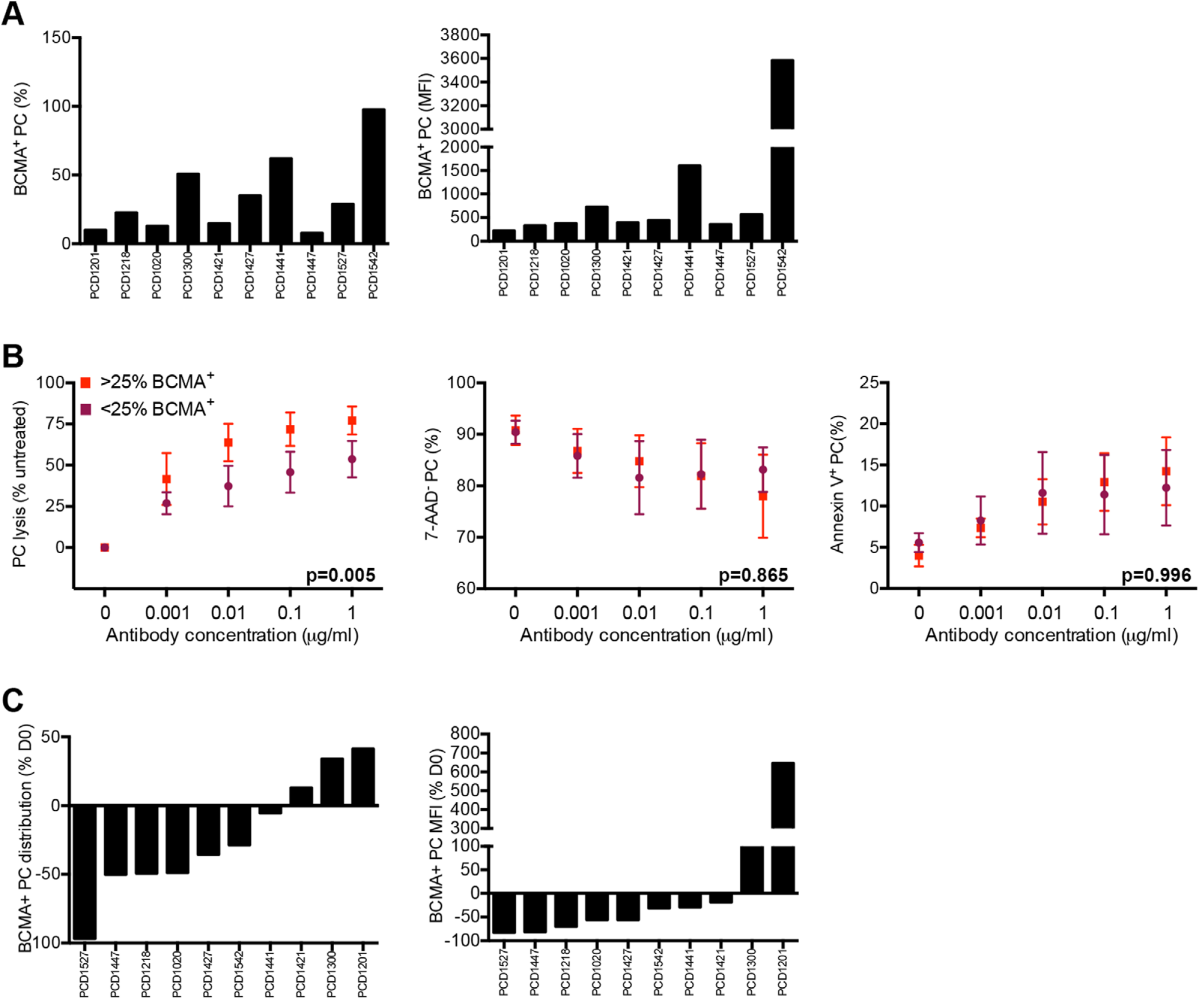
BCMA expression and TNB‐383B anti‐PC activity ex vivo. **A,** B‐cell maturation antigen expression among the 10 bone marrow aspirates tested at baseline following BMMC isolation. Frequency of BCMA+ (%) and mean fluorescence intensity of BCMA+ PC (MFI) were determined by flow cytometry. **B,** Median BCMA+ PC counts (25%) was used as a cutoff value to test the effect of target expression on TNB‐383B‐induced PC lysis, viability (7‐AAD^−^) and death by apoptosis (Annexin‐V^+^) by factorial ANOVA. **C,** Alterations on BCMA expression by PC (frequency of BCMA^+^ PC and BCMA MFI) among the 10 BMMC preparations tested following 24 ± 2 h ex vivo treatment with 1 μg/mL TNB‐383B

BCMA expressing PC were contained in all BMMC specimens, with frequency ranging from 7.7% to 97.6% of total PC content (Figure 2A). Level of BCMA expression also varied greatly as shown by BCMA staining intensity ranging from 217 to 3583. There was no association between BCMA expression and lines of therapy, drugs regimen(s), or disease status.

Median % BCMA expressing PC count was used as a cutoff value to test the effect of target expression on main effect and dose response to TNB‐383B by factorial ANOVA. After applying this cutoff, BCMA^high^ BMMC specimens content had 54.8±27.3% BCMA^+^ PC (BCMA MFI: 332.6±69.4) and BCMA^low^ BMMC specimens had 13.5±5.7% BCMA^+^ PC (BCMA MFI 332.6 ± 69.4). Bone marrow specimens containing high BCMA^+^ PC counts showed increased PC lysis by TNB‐383B (*P* = .005) confirming specificity of BCMA‐targeted PC killing by the T‐BsAb (Figure 2B). No significant changes were observed on remaining PC viability or death by apoptosis after 24 h of treatment, regardless of which dose of antibody was applied.

At its highest tested dose (1 μg), TNB‐383B reduced % BCMA+ PC in 7/10 BMMC specimens; with the remaining samples displaying an increase instead (Figure 2C). In a specimen obtained from patient previously treated with daratumumab, almost complete elimination of target cells (96.8% reduction) was observed. Although BCMA staining intensity diminished in 8 of 10 specimens upon TNB‐383B exposure (Figure 2C), none had complete abrogation of target expression (BCMA MFI loss ranging – from −82.6% to ‐18.3%).

#### 3.3.1 TNB‐383B promotes CTL degranulation

**FIGURE 3:**
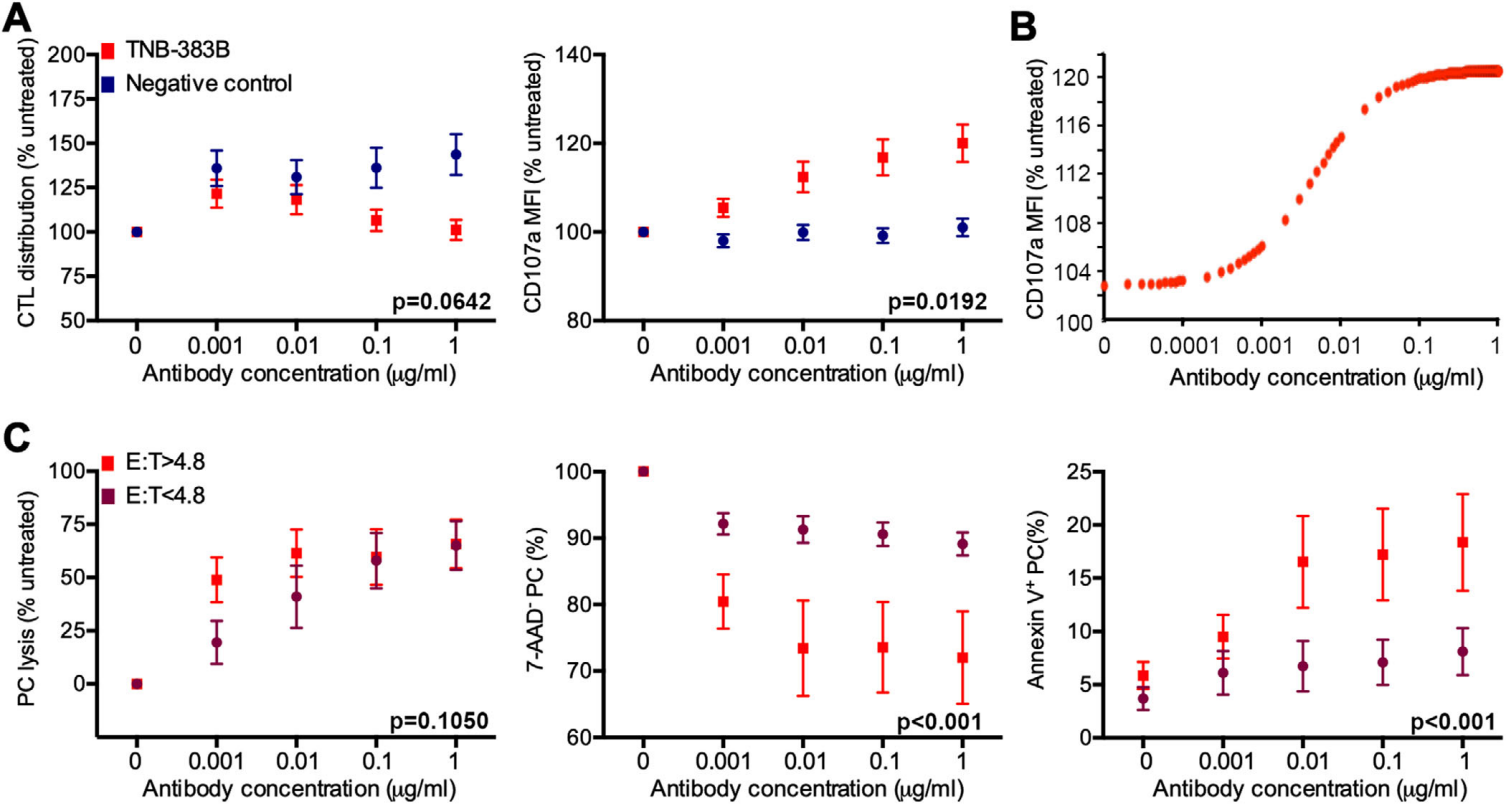
TNB‐383B pro‐CTL function activity ex vivo. A, Interaction of treatment analysis to determine the impact of treatment (TNB‐383B vs negative control) and dose (0.001 to 1 μg) on CTL distribution and degranulation (CD107a expression). Tukey's sequential trend test was performed for each experimental treatment group, and each variable tested, utilizing ANOVA models and contrast statements to detect a linear trend in experiments involving increasing doses. B, Dose‐dependent CTL degranulation (CD107a MFI normalized to untreated) induced by TNB‐383B using E_max_ parametric model to estimate dose response curves, interpolating between tested doses. C, Median E:T ratio (4.8) was used as a cutoff value to test the effect PC and CTL relative distributions on TNB‐383B‐induced PC lysis, viability (7‐AAD^−^) and death by apoptosis (Annexin‐V^+^) by factorial ANOVA

Cytotoxic T lymphocyte distribution (% CTL) and degranulation (CD107a MFI) was quantified by flow cytometry following treatment with TNB‐383B or negative control (24 ± 2 h incubation at doses ranging from 0.001 to 1 μg). At the highest dose tested, TNB‐383B did not alter CTL distribution but BMMC treated with negative control (containing an anti‐CD3 arm but lacking specificity for BCMA) tended to show signs of CTL expansion without reaching statistical significance (101.2 ± 36.4% vs 143.7 ± 36.4% respectively, *P* = .0642; Figure 3A). CTL degranulation on the other hand did vary between the two treatment groups with TNB‐383B promoting CD107a expression by CTL in a dose‐dependent fashion while the negative control treatment failed to do so (*P* = .0192). E_max_ parametric modeling showed that CTL degranulation was triggered by TNB‐383B at doses as low as 0.001 μg (Figure 3B).

Relative T cell distributions in BMMC preparations did not directly correlate with PC lysis or viability post‐TNB‐383B treatment. E:T ratio on the other hand was associated with PC killing/apoptosis by TNB‐383B (Figure 3C). Median E:T ratio (4.8) was used as the cutoff value to test the effect of E:T ratio on main effect and dose response to TNB‐383B by factorial ANOVA. Bone marrow specimens containing high E:T ratios (32.2 ± 14.5) treated with TNB‐383B reached lower viable and higher apoptotic PC counts compared with specimens with low E:T ratio (2.2 ± 0.6; *P* < .001).

#### 3.3.2 TNB‐383B induces a mild BMMC cytokine response ex vivo

**FIGURE 4:**
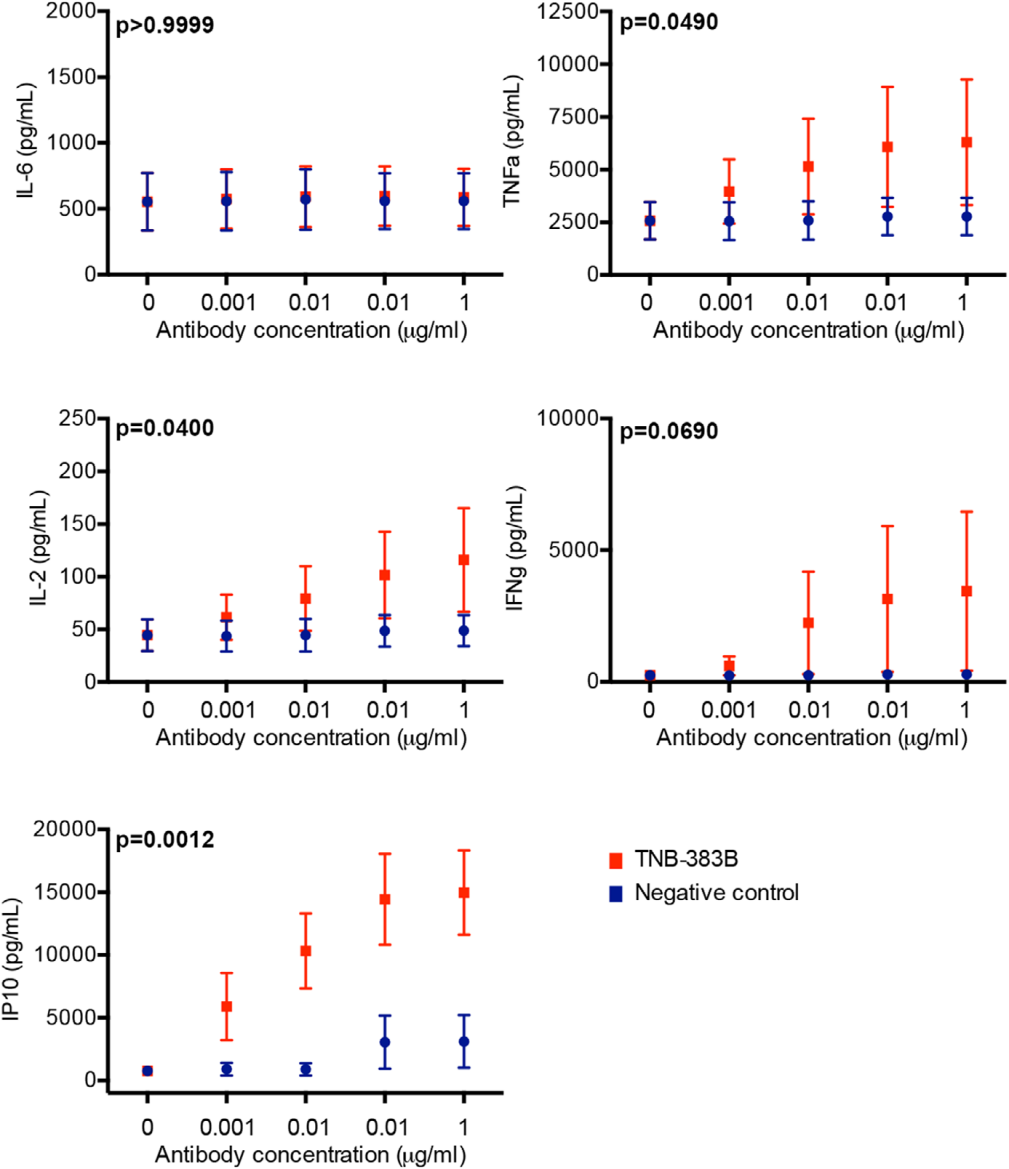
Cytokine response to TNB‐383B ex vivo. Interaction of treatment analysis to determine the impact of treatment (TNB‐383B vs. negative control) and dose (0.001 to 1 μg) on cytokine response after 24 h of treatment with TNB‐383B. Tukey's sequential trend test was performed for each experimental treatment group, and each variable tested, utilizing ANOVA models and contrast statements to detect a linear trend in experiments involving increasing doses

Among the 27 variables tested to assess cytokine response to TNB‐383B, 3 (IL‐2, TNF‐α, and IP10) were differentially modulated between the T‐BsAb or negative control treatment (*P* < .05; Figure 4). IL‐2 and TNF‐α concentrations were highly heterogeneous among untreated BMMC cultures. Upon exposure to TNB‐383B, IL‐2/TNF‐α response displayed a bimodal distribution with five BMMC specimens lacking cytokine modulation and the remaining five a moderate increase (IL‐2: 1.3 ± 0.2 vs 6.6 ± 3.3‐fold change, *P* < .001; TNF‐α: 1.4 ± 0.2 vs 6.0 ± 3.1‐fold change, *P* < .001). This pattern was independent of BMMC culture content (PC BCMA expression or E:T ratio), number of lines of therapy, prior drug regimens, or whether patients were on active treatment. IP‐10 concentrations were also heterogeneous at baseline among BMMC cultures (766.4 ± 348.3 pg/mL) and this chemokine was highly modulated, in a dose‐dependent fashion, by TNB‐383B (*P* < .0001) with an average increase of 50 ± 15.43 at maximum tested antibody dose.

Additional CRS associated markers such as IL‐6 and IFN‐γ were tested (Figure 4). IL‐6 was highly heterogeneous among untreated BMMC cultures. E:T ratio was the main driver of this heterogeneity with specimens containing high E:T ratio (>4.8) producing more IL‐6 compared with E:T low specimens (IL‐6: 952.1 ± 354.4 vs 87.4 ± 76.3; *P* < .01). The T‐BsAb did not significantly alter IL‐6 production even at the highest dose tested. IFN‐γ production was only triggered by TNB‐383B in three of seven BMMCs cultures tested (IFN‐γ: 30 ± 11.7‐fold increase in average at 1 μg compared with untreated) and was not significantly modulated compared with negative control (*P* = .0690). Four additional cytokines (IL‐12p70, IL‐13, IL‐17, RANTES) were also marginally modulated by TNB‐383B without reaching statistical significance (*P* = .6819, *P* = .5123, *P* = .5956, and *P* = .5207, respectively).

## 4 DISCUSSION

Harnessing and enhancing T cell‐mediated eradication of cancer represents an attractive therapeutic avenue in the management of MM. Numerous formats of T cell engaging BsAb are being clinically developed. TNB‐383B, a BCMA‐targeting T‐BsAb, is uniquely designed to elude systemic T cell activation and minimize cytokine release syndrome [7]. To test for its potential use in targeted T‐cell immunotherapy, we investigated ex vivo efficacy of TNB‐383B. We used flow cytometry based assays in BMMC, in which TNB‐383B dependent engagement of autologous BM T cell to mediate lysis of primary MM cells can be directly measured, thus mimicking the target as well as effector cells diversity seen in patient‐derived bone marrows. In terms of activity, TNB‐383B induced lysis of primary MM cells by autologous T cells was observed at dose as low 0.001 μg, reaching 65% reduction of total PC after 24 h even in aspirates with a very low E:T ratio.

The limitations of the study are a small sample size its focus and relapsing MM patients rather than new diagnoses. In addition, prior line of therapy(ies) and cytogenetics were very heterogeneous. Yet, BCMA expression on PC was observed in all bone marrow samples, implying that BCMA expression is retained with subsequent relapses and persists in the residual MM cells after treatment, thus providing the rationale for maintenance strategies targeting BCMA. Although primary MM cells varied in BCMA expression, with BCMA^+^ PC content ranging from 10% to 100% and a 1 log variation in BCMA MFI, we observed a dose‐dependent PC lysis by TNB‐383B in all specimens tested, the rate of which was partially determined by BCMA expression level. While demonstrating BCMA‐specific PC lysis, preclinical studies with other T‐BsAb such as EM801 or JNJ‐957 have not established correlation between PC lysis and BCMA‐binding capacity or expression [8, 9]. Similarly, early clinical data with CC‐93269 (previously known as BCMA0TCB2/EM901), a high BCMA‐affinity T‐BsAB derived from EM801, showed neither soluble BCMA concentration nor density of BCMA expression in the surface of MM cells correlated with clinical response [10].

By using BMMC we captured the E:T ratios reflecting the high heterogeneity in T cell numbers found in the bone marrow of patients with MM. TNB‐383B induced PC lysis, regardless of very high or low E:T ratio. When comparing bone marrow aspirates with high vs low E:T ratios (median 8.0 [range, 6‐8] versus 2.0 [range, 1‐3]), we showed that higher E:T ratio was associated with low viability and high PC apoptotic rate following 24 h TNB‐383B treatment. In line with our observation, ex vivo testing of EM801 showed no significant differences in PC lysis in bone marrow aspirates with high versus low E:T ratio (median 2.9 [range, 0.2‐21] vs 1.0 [range, 0.03‐4.3]) following 48 h T‐BsAb treatment [8]. On the other hand, in vitro testing of BI836909 (AMG 420) using MM.1R multiple myeloma cells and purified T cells, using E:T ratio ranging from 1:100 to 10:1, showed maximal PC lysis at E:T ratio of 1:1 or higher with EC50 values decreasing with increasing E:T cell ratios [11].

Compared with anti‐BCMA CAR T cells, early clinical studies with BCMA‐targeting T‐BsAb show lower rates of CRS [12, 13, 14]. T cell engagement by the αCD3 moiety of T‐BsAb is a key driver of toxicity [15]. Upon recognition of the target tumor antigen, T cell activation and expansion can result in CRS characterized by markedly elevated soluble IL2, IL6, IL10, and IFN‐γ. In this study, TNB‐383B induced dose‐dependent CTL degranulation with no T cell expansion, a mild increase (<100‐fold) of CRS‐associated cytokines (IL‐2, TNF‐α) and no increased IL‐6 production in BM aspirate culture supernatants. These findings are consistent with previous in vitro work using PBMCs with this T‐BsAb showing that its αCD3 moiety preferentially engages effector over regulatory T cells and have low T‐activating activity [6, 7]. Critically, E_max_ parametric modeling in our ex vivo assay showed that maximum PC lysis by TNB‐383B was reached at 0.01 μg while maximum CTL degranulation required up to 0.1 μg dose, confirming that full T cell activation is not required to achieve killing of target cells. By contrast, in vitro studies with a BCMA‐targeting BiTe^®^ construct BI836909 (AMG420) showed dose‐dependent T cell degranulation (CD107a expression) and ≥1000 fold increase of CRS associated cytokines in PBMC culture supernatants [11]. Compared with these BCMA‐targeting BsAb constructs, TNB‐383B stimulates very low levels of cytokine release, but drives robust tumor antigen‐specific killing ex vivo, underpinning a next‐generation of T‐BsAb platform in which potent cytotoxicity is uncoupled from high levels of cytokine release, with a potential for a wider therapeutic window in the clinic.

Beyond CRS‐associated analytes, TNB‐383B did not significantly alter chemokine and growth factor production at the tested doses except for near 1500‐fold induction of chemokine interferon gamma‐induced protein 10 (IP10). IP‐10 is a C‐X‐C motif chemokine 10 (CXCL10) that often acts as a chemoattractant for T cells [16]. IP10 was produced reproducibly in a dose‐dependent fashion in our assay, but we show no correlation with IFN‐γ production or with PC lysis. Overall, these findings suggest that paracrine stimulation through IFN‐γ or PC stimulation is an unlikely source of IP10 production. A recent study of an anti‐CD3 x anti‐EGFR T‐BsAb against locally advanced or metastatic pancreatic cancer showed this construct promotes serum IP10, suggesting that T cell engagement may lead to IP10 production [17]. An in vivo study in mice (confirmed with human PBMCs) has shown that IP10 is produced by CD8 T cells in response to CD27/CD70 co‐stimulation [18]. IP10 is a chemotactic factor for CXCR3+ T cells (mostly TH1 and CTLs) and CXCR3+ NK cells [19]. It can also alter NK cell behavior in multiple myeloma by increasing NKp30 expression by CD56^low^CD16^low^ NK cells and potentially impeding their cytolytic activity [20]. Interestingly, immune modulatory drugs (IMiDs, Pomalidomide, Thalidomide) have shown to strongly suppress TLR4‐induced IP10 production by peritoneal macrophages in a mouse model suggesting a potential for synergy with TNB‐383B [21].

In summary, our evaluation of TNB‐383B indicates potent ex vivo target cell killing through endogenous T cell engagement with attenuated cytokine release, which is likely to be critical for clinical tolerability and efficacy. Complementing this effort, a phase I open‐label study (NCT03933735) is currently evaluating the safety and clinical activity of TNB‐383B in patients with relapsed and refractory MM.

## AUTHOR CONTRIBUTIONS

Research design: D.F., M.B., S.U.; Protocol development: D.F., N.S., F.G., D.P., B.B.; Tissue procurement and patient consent: A.N., E.T.; Reagent supply: D.P., B.B.; Lab work: D.F., F.G., K.R., M.L.; Data analyzes: D.F., M.B., M.R., J.S.; Manuscript preparation: D.F., M.B., B.P., S.A., P.V., S.U.

## CONFLICT OF INTEREST

D.F. received research funding from TeneoBio; M.B. served on speaker bureau for Amgen, BMS and Takeda; consultant for Sanofi Genzyme; received research funding from Janssen, MedImmune, Takeda and Prothena. D.P. and B.B are employees of TeneBio; B.P. was formally employed by Bristol‐Myers Squibb; S.A. received research funding from Mundipharma‐EDO, consulted for Celgene, served in advisory committee for Takeda, Celgene, Amgen, Sanofi, Karyopharm; P.V. served on speaker bureau of Celgene, Janssen, Takeda, served in advisory committee for Amgen, Celgene, Janssen; S.U. received research funding from Array BioPharma, Amgen. Celgene, Onyx, Sanofi, Janssen, Pharmacyclics, Bristol‐Myer‐Squibb, Seattle Genetics, SkylineDX, TeneoBio, served on speaker bureau for Amgen, Celgene, Takeda, Janssen, served in advisory committee for Abbvie, Amgen, Celgene, GSK, BMS, Sanofi, SkylineDX, Takeda, Seattle Genetics, Janssen.

## Supporting information

Supporting information
