# Supplementary material for: Ex vivo efficacy of BCMA‐bispecific antibody TNB‐383B in relapsed/refractory multiple myeloma
Source: EJHaem. 2020 Aug 1;1(1):113–21. doi: 10.1002/jha2.69 (PMC9175895; doi:10.1002/jha2.69)
Supplement: Supplementary file 1 — Supporting information [file JHA2-1-113-s001.pdf]

**SUPPLEMENTAL MATERIAL**

**Table S1: Antibody used flow cytometry-based ex vivo BsAb antibody assay**

| Marker | Channel | Clone | Supplier | Cat # |
|---|---|---|---|---|
| CD38 | FITC | Multi-Epitope | ALPCO | 70-38F2 |
| CD269 (BCMA) | PE | 19F2 | Biolegend | 357504 |
| CD45 | PE-CF594 | HI30 | BD Biosciences | 562279 |
| 7-AAD | PerCP-Cy5.5 | Kit | BD Biosciences | 559925 |
| CD8 | PE-Cy7 | RPA-T8 | BD Biosciences | 557750 |
| Annexin V | APC | Kit | BD Biosciences | 550474 |
| CD3 | APC-C750 | UCTH-1 | ALPCO | 70-3AC750 |
| CD138 | BV421 | MI15 | BD Biosciences | 562935 |
| CD107a | BV510 | H4A3 | BD Biosciences | 563078 |

**Figure S1: Gating strategy for plasma cell and cytotoxic T cells.** Following (**A**) doublet & debris exclusion based on morphological features, (**B**) plasma cells were defined as CD38$^{high}$/CD138$^{high}$/CD45$^{-/int}$ and PC lysis was calculated for each treatment group as follow [$\% \ lysis = \frac{Treated\ PC\ count \times 100}{Untreated\ PC\ counts}$]. (**C**) Cytotoxic T cells were defined as CD3$^{+}$/CD8$^{+}$/CD45$^{high}$ (**C**) and CTL distribution was calculated for each treatment group as follow [$\% \ CTL = \frac{Treated\ CTL\ count \times 100}{Untreated\ CTL\ counts}$].

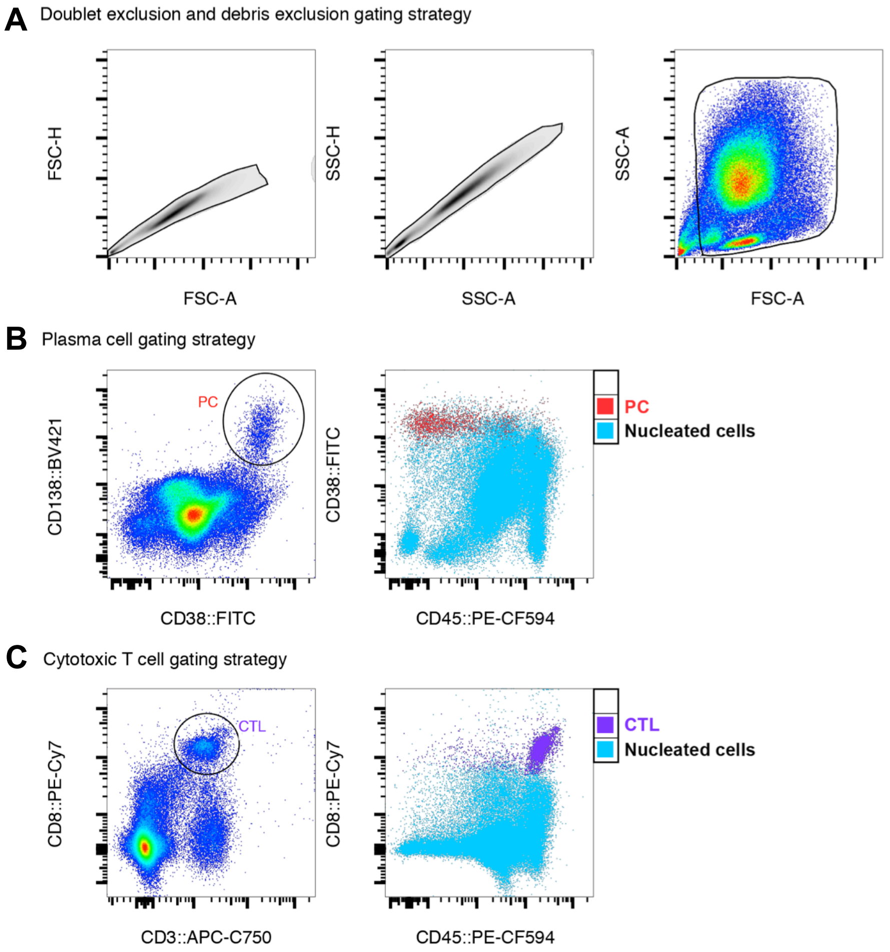

**Figure S2: Gating strategy for plasma cell and cytotoxic T cells phenotyping.** **(A)** Gated from $CD38^{high}/CD138^{high}/CD45^{-/int}$ population, live plasma cells were defined for each treatment group as 7-AAD$^{-}$ PC and dead apoptotic cells as 7-AAD$^{+}$/Annexin V$^{+}$ PC. (**B)** Gated from $CD38^{high}/CD138^{high}/CD45^{-/int}$ population, BCMA expression was expressed for each treatment group as average frequency of PC expressing BCMA (PC BCMA$^{+}$) and mean fluorescence intensity (BCMA MFI). BCMA expression by T cells (defined as $CD3^{+}/CD45^{high}$ population) was used as negative control. (**C**) Gated from $CD3^{+}/CD8^{+}/CD45^{high}$, CTL degranulation was determined for each treatment group by calculating mean fluorescence intensity of CD107a.

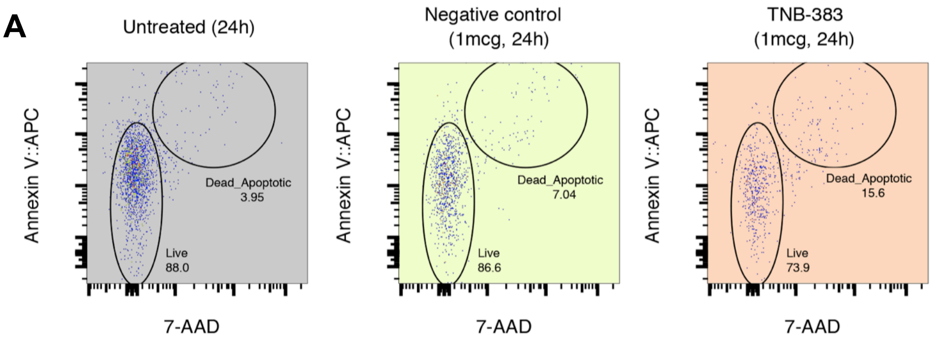


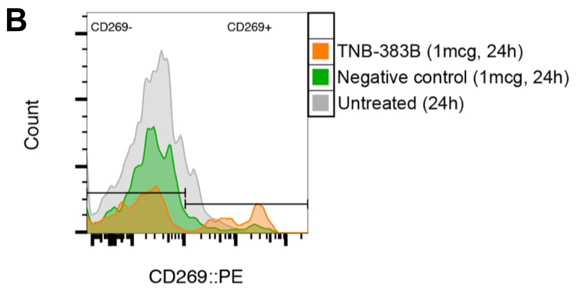


BCMA expression

| | |
|---|---|
| Untreated | MFI : CD269 : 314 |
| Negative control | MFI : CD269 : 285 |
| TNB-383 | MFI : CD269 : 306 |

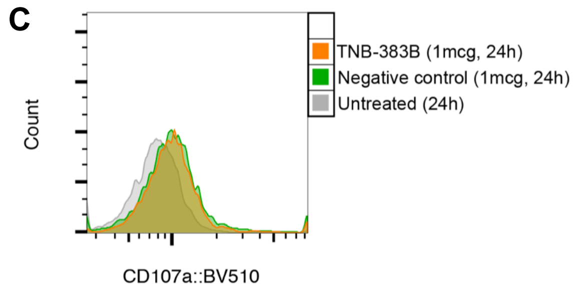


CTL degranulation

| | |
|---|---|
| Untreated | MFI : CD107a : 78.9 |
| Negative control | MFI : CD107a : 98.4 |
| TNB-383 | MFI : CD107a : 98.2 |
